# RodZ and PgsA Play Intertwined Roles in Membrane Homeostasis of *Bacillus subtilis* and Resistance to Weak Organic Acid Stress

**DOI:** 10.3389/fmicb.2016.01633

**Published:** 2016-10-21

**Authors:** Johan van Beilen, Christoph J. Blohmke, Hendrik Folkerts, Richard de Boer, Anna Zakrzewska, Wim Kulik, Fred M. Vaz, Stanley Brul, Alexander Ter Beek

**Affiliations:** ^1^Laboratory for Molecular Biology and Microbial Food Safety, Swammerdam Institute for Life Sciences, University of AmsterdamAmsterdam, Netherlands; ^2^Laboratory Genetic Metabolic Diseases, Academic Medical Center, University of AmsterdamAmsterdam, Netherlands

**Keywords:** weak organic acids, acetic acid, sorbic acid, *Bacillus subtilis*, rod Z, pgs A, membrane compositional fluctuations

## Abstract

Weak organic acids like sorbic and acetic acid are widely used to prevent growth of spoilage organisms such as Bacilli. To identify genes involved in weak acid stress tolerance we screened a transposon mutant library of *Bacillus subtilis* for sorbic acid sensitivity. Mutants of the *rodZ* (*ymfM*) gene were found to be hypersensitive to the lipophilic weak organic acid. RodZ is involved in determining the cell’s rod-shape and believed to interact with the bacterial actin-like MreB cytoskeleton. Since *rodZ* lies upstream in the genome of the essential gene *pgsA* (phosphatidylglycerol phosphate synthase) we hypothesized that expression of the latter might also be affected in *rodZ* mutants and hence contribute to the phenotype observed. We show that both genes are co-transcribed and that both the *rodZ*::mini-Tn*10* mutant and a conditional *pgsA* mutant, under conditions of minimal *pgsA* expression, were sensitive to sorbic and acetic acid. Both strains displayed a severely altered membrane composition. Compared to the wild-type strain, phosphatidylglycerol and cardiolipin levels were lowered and the average acyl chain length was elongated. Induction of *rodZ* expression from a plasmid in our transposon mutant led to no recovery of weak acid susceptibility comparable to wild-type levels. However, *pgsA* overexpression in the same mutant partly restored sorbic acid susceptibility and fully restored acetic acid sensitivity. A construct containing both *rodZ* and *pgsA* as on the genome led to some restored growth as well. We propose that RodZ and PgsA play intertwined roles in membrane homeostasis and tolerance to weak organic acid stress.

## Introduction

Weak organic acids (e.g., sorbic-, acetic-, and benzoic- acid) are commonly used preservatives in the food industry since they inhibit the growth of spoilage bacteria, yeasts, and molds (Brul and Coote, 1999; Davidson and Harrison, 2002; Beales, 2004; Brul and Ter Beek, 2010). The acids are most effective at pH conditions close to or below their *pK*_a_ value. Depending on the lipophilic nature of the compound, the neutral undissociated form of the molecule is able to dissolve in and diffuse over the membrane. The hydrophobic tail of, e.g., lipophilic sorbic acid, can also more permanently insert into the membrane perturbing its structure and interfering with the function of proteins (Sheu and Freese, 1972; Stratford and Anslow, 1998; Chu et al., 2009). Inside the cell the acid dissociates and releases protons to a large extend, since most microorganisms exhibit an intracellular pH (pH_i_) near neutrality. Consequently, the proton gradient dissipates and, depending on the buffering capacity of the cell, the cytosol may acidify, affecting oxidative phosphorylation, the transport of nutrients, and a number of other metabolic functions (Bauer et al., 2003; Cotter and Hill, 2003; Brul and Ter Beek, 2010; van Beilen et al., 2014). The generation of reactive oxygen species has been described in *Saccharomyces cerevisiae* and recently has been detected in *Bacillus cereus* upon weak acid stress (Piper, 1999; Mols et al., 2010), which could damage iron-sulfur clusters, proteins, and DNA. Finally, it has been shown that the accumulation of the anion in the cell can cause a rise in osmolarity and affect cytosolic enzymes (Azukas et al., 1961; York and Vaughn, 1964; Russell, 1992).

*Bacillus subtilis* is one of the organisms that causes food spoilage and its growth is inhibited by weak organic acids (Eklund, 1983). Previously, we preformed time-resolved transcriptome analysis of *B. subtilis* sub-lethally stressed with potassium sorbate (KS) to elucidate the sorbic acid adaptive responses of this organism at the molecular level (Ter Beek et al., 2008). The results indicated that sorbic acid induces responses normally seen upon nutrient limitation and alters the expression of many cell envelope-related genes. Upregulation of fatty acid biosynthesis (*fab*) genes and BkdR-regulated genes indicated the synthesis of longer and more branched lipids. We proposed an adaptation in the fatty acid composition of the *B. subtilis* plasma membrane as a stress response mechanism, since the sensitivity of cells toward the *fab* inhibitor cerulenin was reduced in the presence of sorbic acid (Ter Beek et al., 2008). Gene groups regulated by extracytoplasmic function sigma factors SigW and SigX (controlling functions associated with the cell surface and transport) (Mascher et al., 2007) were downregulated, indicating a reduction in cell envelope remodeling and, consequently, a change in cell envelope composition. In addition, similar analyses have been performed by us in later experiments for acetic acid stressed cells in comparison to sorbic acid and the classical uncoupler carbonyl cyanide-m-chlorophenyl hydrazone (CCCP). It was observed that the inhibitory effect of sorbic acid seems to be more focussed on the cell membrane than that of acetic acid and that sorbic acid has an effect on cell physiology that is more akin to a classical uncoupler (Ter Beek et al., 2014; van Beilen et al., 2014). In *B. subtilis*, PgsA is an essential protein committed to the synthesis of phosphatidylglycerol (PG), which besides being the only essential phospholipid is also the precursor for cardiolipin (CL) and lysyl-phosphatidylglycerol (L-PG) (Matsumoto et al., 2006; Salzberg and Helmann, 2008). Lopez and co-workers have shown that salt-stressed cells increase their CL phospholipid levels and decrease both PG and L-PG levels (López et al., 1998). Sorbic acid was shown to interact with the phospholipid headgroups (Chu et al., 2009) and mutants resistant to uncouplers were found to contain mutations in desaturase, resulting in more unsaturated fatty acids (Krulwich et al., 1990). This further corroborates the notion that the membrane is likely to play a crucial role in weak organic acid sensitivity, especially so since this may help to maintain the proton motive force.

A few years ago, RodZ was discovered as new player in bacterial cell morphogenesis (Gerdes, 2009). RodZ has been described in, among others, *Escherichia coli, Caulobacter crescentus* and *B. subtilis* and is reported to interact with the bacterial actin-like MreB cytoskeleton controlling cell shape and cell wall synthesis (Shiomi et al., 2008; Alyahya et al., 2009; Bendezú et al., 2009; White et al., 2010; Dempwolff et al., 2011). Interestingly, in several *Bacillus* species *rodZ* (*ymfM*) lies upstream in the genome of *pgsA*, encoding phosphatidylglycerol phosphate synthase. Until now, no functional link between RodZ and phospholipid synthesis has been reported. But, since RodZ is also associated with the cell elongation complex (den Blaauwen et al., 2008; Alyahya et al., 2009) a link between the only essential phospholipid and the cell envelop seems obvious as they would need to keep track of each other within a growing cell. Moreover, weak organic acids can act as uncouplers, they may interfere with correct localization of several components of the cytoskeleton (Strahl and Hamoen, 2010; van Beilen et al., 2014).

To elucidate novel weak acid resistance mechanisms in *B. subtilis* we created a mini-Tn*10* transposon library and screened for sorbic acid hypersensitive mutants. We show that inactivation of *rodZ* by a transposon insertion and reduction in *pgsA* expression using a conditional mutant lead to a weak acid hypersensitive phenotype. We demonstrate that PgsA depletion contributes primarily to the weak acid sensitivity observed and speculate on a possible link between membrane and cell wall homeostasis through RodZ and PgsA.

## Materials and Methods

### Strains, Growth Conditions, and Genetic Manipulation

All bacterial strains and plasmids used in this study are listed in **Table 1**. The *B. subtilis* strains are derivatives of the laboratory 168 wild-type (WT) lab-strains PB2 (*trp2C*) or 1A700 (*trp2C*). *E. coli* strains XL1-Blue and MC1061 were grown in lysogeny broth (LB) at 37°C. *B. subtilis* strains were grown in LB, buffered with 80 mM 3-(*N*-morpholino)propanesulfonic acid (MOPS) at pH 7.4 or 6.4 at 37°C. When required for selection, the following antibiotics were added to the medium at given concentrations: 100 μg/ml ampicillin, 1 μg/ml erythromycin, 100 μg/ml spectinomycin, 10 μg/ml kanamycin.

**Table 1:** Plasmids and strains used in this study.

Strain or plasmid	Genotype or description^a^	Source or reference
***Bacillus subtilis* strains**		
1A700	*trp*2C; 168 wild-type	BGSC^b^
PB2	*trp*2C; 168 wild-type	Boylan et al. (1992)
ATB012	*rodZ*::mini-Tn*10*; Sp^R^ (PB2)	This work
MHB001	*pgsA*::P*spac-pgsA*; Em^R^ (1A700)	Hashimoto et al. (2013)
***Escherichia coli* strains**		
XL1-Blue	Cloning host	Stratagene
MC1061	Cloning host	Casadaban and Cohen (1980)
**Plasmids**		
pIC333	mini-Tn*10 tnpA*; Em^R^, Sp^R^	Steinmetz and Richter (1994)
pDG148	P*spac*-MCS; Ap^R^, Ph^R^, Km^R^	Stragier et al. (1988)
pDG-*rodZ*	P*spac*-*rodZ*; Ap^R^, Ph^R^, Km^R^ (pDG148)	This work
pDG-*pgsA*	P*spac*-*pgsA*; Ap^R^, Ph^R^, Km^R^ (pDG148)	This work
pDG-*rodZ-pgsA*	P*spac*-*rodZ-pgsA*; Ap^R^, Ph^R^, Km^R^ (pDG148)	This work

*^a^Sp^*R*^, spectinomycin resistance; Em^*R*^, erythromycin resistance; Ap^*R*^, ampicillin resistance; Ph^*R*^, phleomycin resistance; Km^*R*^, kanamycin resistance; MCS, multiple cloning site.*

*^b^Bacillus Genetic Stock Center (http://www.bgsc.org/).*

Standard molecular genetics techniques were used as described by Sambrook et al. (1989). The pDG148 vector was used to overexpress *rodZ* and *pgsA* (Stragier et al., 1988). The *rodZ* gene was PCR amplified from *B. subtilis* PB2 genomic DNA using the rodZ_FW and rodZ_RV primers (see Supplementary Table S1 of the Supporting Information for the sequences of all used primers). All cloning PCR reactions were performed with *Pfu* polymerase (Fermentas, Thermo Fisher Scientific). The forward primers for each construct contain a ribosome binding site, which is not present on the plasmid when it is cut with *Hin*dIII. The PCR product and vector were digested with *Hin*dIII and *Sal*I and ligated with T4 ligase (Fermentas), thus creating pDG-rodZ. For pDG-pgsA, the *pgsA* gene was amplified using the pgsA_FW and pgsA_RV primers and introduced between the *Hin*dIII and *Sal*I sites of pDG148. The combined rodZ-pgsA construct was amplified using the rodZ_FW and pgsA_RV primers and inserted as described above to create pDG-rodZ-pgsA. All constructs were first transformed to chemically competent *E. coli* MC1061 cells before plasmids were isolated and transformed to competent *B. subtilis* strains. Plasmids were isolated using a QIAprep Spin Miniprep Kit (Qiagen). Competent *B. subtillis* cells were obtained and their transformations were performed as described previously (Kunst and Rapoport, 1995). The nucleotide sequence of all newly constructed plasmids was verified by sequencing.

### Identification of Sorbic Acid-Susceptible Genes

Two independent transposon mutant libraries in *B. subtilis* WT strain PB2 were created as previously described using the mini-Tn*10* delivery vector pIC333 (Steinmetz and Richter, 1994). The resulting libraries were validated for correct transposition efficiency and randomness of transposition (data not shown). Both constructed mutant libraries have the expected statistical properties according to Petit et al. (1990) and Maguin et al. (1992). The two transposon mutant libraries were subjected to a screen to identify mutants hypersensitive to the presence of sorbic acid. The mutant libraries were plated on 80 mM MOPS-buffered LB agar (pH 7.4). Following overnight incubation at 37°C, cells were transferred by replica plating using sterile velvets to 80 mM MOPS-buffered LB agar of pH 6.4, containing 30 mM potassium sorbate (KS). Next, the same sterile velvets were used to replicate cells on LB plates without KS to verify the successful transfer of cells. The mutants that showed no or minimal growth on KS containing LB agar compared to the plates without sorbate were stored at -80°C until further analysis. Chromosomal DNA from the hypersensitive mutants was isolated using the DNeasy Blood & Tissue Kit (Qiagen). The purified DNA was digested with *Hin*dIII. DNA from the restriction reaction was self-ligated using a Ready-To-Go DNA T4 Ligase (Amersham Bioscience). Next, ligated DNA was used directly for transformation of *E. coli* XL1-BLUE cells (Stratagene). Isolated plasmid DNA was used as a template for sequencing with specific to either end of the transposon and enabling sequencing of the cloned chromosomal DNA. The primers were purchased from Isogen Life Science (see Supplementary Table S1 of the Supporting Information for the sequences) and the sequencing was performed by BaseClear. The sequencing results were aligned to the re-annotated genome sequence of *B. subtilis* 168 using BLAST at the SubtiList database (Barbe et al., 2009)^1^ in order to identify the affected gene.

### Phase-Contrast Microscopy

*Bacillus subtilis* strains PB2 (WT) and *rodZ* transposon mutant ATB012 were grown into the exponential phase in 80 mM MOPS-buffered LB medium of pH 6.4. Cells were immobilized on 1% agarose as described previously by Koppelman et al. (2004), and photographed with a CoolSnap *fx* (Photometrics) CCD camera mounted on an Olympus BX-60 fluorescence microscope through an UPLANFl 100x/1.3 oil objective (Japan).

### Characterization of Stress Sensitivity of Various Mutant Strains

To further characterize the acid sensitivity of the (transposon) mutants the cells were grown on both solid agar plates and in liquid medium containing different concentrations of KS, potassium acetate (KAc) or NaCl. For the conditional *pgs*A mutant strain (P*spac*-*pgsA*) (Hashimoto et al., 2013), the medium contained additional 0.1 mM isopropyl-β-D-1-thiogalactopyranoside (IPTG). Overexpression of *rodZ* and/or *pgsA* from the pDG148 vector was induced with 1 mM IPTG in exponentially growing cells 3 h before the start of a stress experiment. For the plating assay on solid medium, the cells were first grown exponentially in 80 mM MOPS-buffered liquid LB medium of pH 6.4, containing the appropriate antibiotics and 0.1 mM IPTG, at 37°C. At an OD_600_ of 0.2, 10-fold serial dilutions of the cultures were spotted on 80 mM MOPS-buffered LB plates of pH 6.4, containing 1% agar and IPTG if required, together with the indicated stress factors. After 24 h of incubation at 37°C pictures of the plates were taken. For the plating assays, the following end-concentrations of chemicals were used: KS: (15, 30, or 40 mM), KAc (80, 125, or 200 mM), and NaCl (0.7 or 1.4 M). Biologically independent experiments were performed at least three times. To monitor the growth of mutants in liquid media, exponentially growing cultures were grown to an OD_600_ of 0.4. Then, the cells were twofold diluted in a 96-well micro titer plate containing different concentrations of weak organic acid or NaCl (see below), and IPTG if required. Cells were further cultivated in a FluoStar Optima microtiter plate reader (BMG Labtech) under rigorous shaking at 37°C for 12 h. Cells were exposed to the following stresses: KS (15 mM or 40 mM), KAc (40 mM or 125 mM), and NaCl (0.7 or 1.4 M). All conditions were tested in the microtiter plate reader at least in duplicate, and biologically independent experiments were performed at least three times.

### Reverse Transcriptase PCR (RT-PCR)

Cells of *B. subtilis* wild-type strain PB2 and *rodZ* transposon mutant strain ATB012 were grown exponentially in 80 mM MOPS-buffered LB medium of pH 6.4. At an OD_600_ of 0.2, half of the cells were exposed to 3 mM of KS and samples were withdrawn from the stressed and control cultures at 0, 10, 20, 30, 45, and 60 min after the addition of KS. Cultures of PB2 harboring empty pDG148 and pDG148-rodZ were grown exponentially in MOPS-buffered LB medium of pH 6.4 and 1 mM IPTG was added 3 h before sampling. At an OD_600_ of 0.2 samples were taken every 30 min for 2 h. All samples were snap-frozen in liquid nitrogen and stored at -80°C prior to RNA extraction. Biological independent experiments were performed twice.

Total RNA was isolated using the RNeasy Kit (Qiagen), as described by the manufacturer’s instructions. Total RNA was eluted in sterile RNase/DNase free water (Ambion), concentrations were determined with Nanodrop UV spectroscopy (Ocean Optics), and integrity was analyzed by agarose gel electrophoresis. RNA samples were treated with Turbo DNase (Ambion) to remove genomic DNA as described by the manufacturer.

To see if *rodZ* and *pgsA* are part of the same transcriptional unit and possibly the same operon, a reverse transcriptase reaction using the Superscript First Strand kit (Invitrogen) was performed with RNA isolated from the WT strain and a single reverse primer annealing to the sequence of *pgsA*, named pgsA_Q1_RV. cDNA was amplified for 40 cycles in a Biometra T3000 Thermocycler using the rodZ_Q1_FW and pgsA_Q1_RV primers. The PCR product was analyzed on a 1% agarose gel. The visible band was isolated from the gel using the QIAquick gel extraction kit (Qiagen) and the DNA was sequenced.

To determine the expression levels of *rodZ* and *pgsA* in the WT and mutant strains cDNA was synthesized using the Superscript First Strand kit (Invitrogen), using random hexamers. Semi-quantitative real-time PCR analysis was carried out on a 7300 Real-Time PCR system (Applied Biosystems). Primer Express 3.0 software (Applied Biosystems) was used to design specific primers (purchased from Isogen Life Science) for real-time PCR (see Supplementary Table S1 of the Supporting Information). Reactions were carried out in a 20 μl mixture consisting of 1 μl of 3 μM specific primers, 5 μl of 100-fold-diluted cDNA template and SYBR green PCR master mix (Applied Biosystems). The cycling conditions were as follows: 1 cycle at 50°C for 2 min, 1 cycle at 95°C for 10 min, and 40 cycles at 95°C for 15 s and at 60°C for 1 min. Melting curves were used to monitor the specificity of the reaction. RNA of all time points and independent experiments were analyzed with real-time PCR in triplicate. Because the amplification efficiencies of the target and reference genes was tested and found to be approximately equal (not shown), the ΔΔ*C_T_* method could be used to calculate relative gene expressions(Livak and Schmittgen, 2001). The *accA* (α subunit of acetyl-CoA carboxylase) and *rpsM* (small subunit ribosomal protein S13) genes were used as two independent internal controls, since their expression levels were stable during exponential growth, irrespective of sorbic acid stress.

### Phospholipid Analysis

Cultures of the different *B. subtilis* strains were grown exponentially in MOPS-buffered LB medium of pH 6.4. At an OD_600_ of 0.2, half of each culture was stressed with 5 mM KS and 45 min later 50 ml of each cell culture was harvested by centrifugation (5 min, 4000 rpm) and the pellets were frozen in liquid nitrogen. Three independent biological replicates were assessed.

For HPLC-MS/MS analysis, samples were lyophilized overnight. The total protein concentration of the samples was measured using a BCA Protein assay kit (Thermo Scientific). Cell material corresponding to 1 mg protein was resuspended in 300 μl water and 300 μl 1:1 chloroform-methanol (v/v). The following internal standards (obtained from Avanti Polar Lipids, Inc.) were added: phosphatidylglycerol (PG), lysyl-phosphatidylglycerol (L-PG), phosphatidylethanolamine (PE), and cardiolipin (CL). Each sample including internal standards was shaken vigorously and placed on ice for 15 min, after which it was centrifuged at 1000 ×*g* for 10 min. The organic layer was transferred into another tube, and the aqueous layer was re-extracted with 3 ml of 2:1 chloroform-methanol (v/v). The combined organic layers were evaporated under a stream of nitrogen at 45°C. The residue was dissolved in 150 μl of 50:45:5 chloroform-methanol-water (v/v/v) (Houtkooper et al., 2006).

The relative abundances of the species in the sample-extracts were determined, using HPLC-MS/MS. The liquid-chromatographic separation was achieved on a modular HPLC system (Surveyor; Thermo Finnigan) consisting of a cooled autosampler set at 12°C, a low-flow quaternary MS pump, and an analytical HPLC column: 2.1 × 250 mm silica column, 5 μm particle diameter (Merck). Phospholipids were separated and eluted with a programmed linear gradient between solution B (chloroform-methanol, 97:3, v/v) and solution A (methanol-water, 85:15, v/v) as described previously (Houtkooper et al., 2006). MS/MS analyses were performed on a TSQ Quantum AM (Thermo Finnigan Corporation, San Jose, CA, USA) operated alternating in the negative- and positive ion electrospray ionization (ESI) mode in consecutive runs. The surface induced collision was set at 10 V; spray voltage was 3600 V and the capillary temperature was 300°C. In the MS/MS experiments argon was used as collision gas at a pressure of 0.5 mtorr; collision energy ranged between 20 and 40 eV for the different optimized transitions. In the negative mode mass spectra of CL molecular species were obtained by continuous scanning between *m*/*z* 400–*m*/*z* 1000 (2 s/scan). In the positive mode characteristic constant neutral loss (CNL) scans were used to selectively detect specific phospholipids in their corresponding retention time windows: CNL(141) for PE, CNL(172) for PG, and CNL(300.1) for L-PG.

## Results

### Identification of *rodZ*::mini-Tn*10* as a Sorbic Acid-Hypersensitive Clone

**FIGURE 1:**
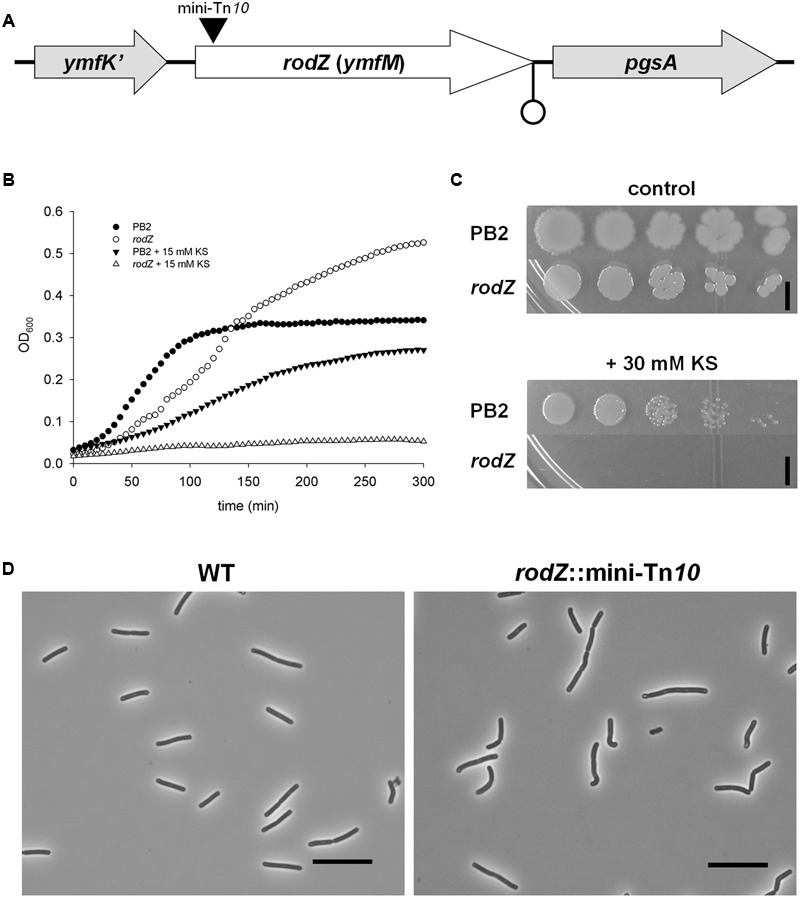
**Inactivation of *rodZ* (*ymfM*) by a transposon leads to a sorbic acid hypersensitive phenotype. (A)** Positioning of the *rodZ* gene between *ymfK’* and *pgsA* in the *Bacillus subtilis* 168 genome. The black triangle indicates the location of the mini-Tn*10* transposon insertion and a possible terminator (due to an inverted repeat) is shown at the end of the *rodZ* sequence. **(B)** Growth curves of WT strain PB2 and the *rodZ*::mini-Tn*10* mutant grown in liquid LB medium of pH 6.4 in control conditions (closed and open circles, respectively) or the presence of 15 mM KS (closed downward triangles and open triangles, respectively). **(C)** Ten-fold dilutions of exponentially growing WT (PB2) and *rodZ* mutant cells were spotted onto LB plates of pH 6.4 containing no (upper) or 30 mM KS (lower). Plates were photographed after 24 h of growth at 37°C. The black bar indicates 5 mm. **(D)** Cell morphology of WT (PB2) and *rodZ*::Tn*10* mutant cells as observed with phase contrast microscopy. Phase contrast micrographs of typical examples of cells grown exponentially in liquid cultures are given. Note the curved rod-shape of the *rodZ*::mini-Tn*10* cells. Scale bar: 10 μm.

In order to identify stress resistance mechanisms against weak organic acids and potential new antimicrobial targets we screened two independent transposon mutant libraries of *B. subtilis* 168 lab-strain PB2 for sorbic acid-hypersensitive clones on LB plates of pH 6.4 containing 30 mM potassium sorbate (KS). Around 10.000 clones were screened for sorbic acid sensitivity and resulted in 132 candidates in an initial evaluation. After thorough investigation of these mutants both on solid and in liquid medium, we ended up with four verified clones displaying a hypersensitive phenotype toward KS. All four clones were identified to have a transposon insertion in the *rodZ* (*ymfM*) gene (**Figure 1A**). The mutants containing an interrupted *rodZ* gene were isolated from both independently constructed transposon libraries and the location of insertion (at basepair 51) was found to be the same for all four mutants.

Sorbic acid hypersensitivity in liquid and on solid media of one of the identified *rodZ*::mini-Tn*10* mutants (strain ATB012) is shown in **Figures 1B,C**, respectively. In non-stressed liquid cultures of pH 6.4 the transposon mutant lagged a bit behind the WT strain PB2, yet it reached a higher yield than the WT strain after 5 h. However, when stressed with 15 mM KS the *rodZ* mutant, unlike the WT strain, displayed only a very slow increase in optical density. Although WT strain PB2 was able to grow, the mutant did not form colonies on LB plates of pH 6.4 containing 30 mM KS after overnight incubation at 37°C (**Figure 1C**, lower). It can be noted that the colonies of mutant strain ATB012 formed on plates are more round when compared to PB2 (**Figure 1C**, upper). Additionally, the *rodZ*::Tn*10* mutant was found to display an irregular cell morphology, having curved endings and being broader when grown in the exponential phase and examined under the microscope, with wild-type cells being 1.03 ± 0.06 μm and *rodZ*::Tn*10* cells 1.13 ± 0.07 μm (**Figure 1D**).

### *rodZ* and *pgsA* Are Co-transcribed

In *B. subtilis* and in most Gram-positive bacterial genomes that have *rodZ*, essential gene *pgsA* lies next to *rodZ* (Alyahya et al., 2009) (**Figure 1A**). We hypothesized that *rodZ* and *pgsA* are part of the same operon, although the two genes are separated by an inverted repeat which, in spite of the lack of a T-rich tail, may act as a termination sequence (Rasmussen et al., 2009). While we cannot rule out their presence, we did not find indications for additional regulatory elements of *pgsA* expression. To confirm that *rodZ* and *pgsA* are indeed co-transcribed, we performed a reverse transcriptase reaction using one single reverse primer annealing to *pgsA* and purified RNA of WT strain PB2 as the template. A PCR reaction using specific primers for *rodZ* and *pgsA* resulted in one specific product. The sequence of the fragment corresponded exactly with the region between *rodZ* and *pgsA* (**Supplementary Figure S1** of the Supporting Information).

Additionally we tested whether the mRNA levels of *rodZ* and/or *pgsA* were affected by sorbic acid stress. Stressing exponentially growing cells of the WT strain in LB medium of pH 6.4 with 3 mM KS did not significantly change the expression levels of both genes during the first 60 min after KS stress (See Supplementary Excel Data Sheet S1). This observation was in line with our previous studies performing microarrays with 3 mM KS-stressed cells in defined minimal medium of pH 6.4 (Ter Beek et al., 2008).

### Stress Profiles of the *rodZ* Transposon Mutant and a Conditional *pgsA* Mutant

RT-PCR results indicated a transcriptional link between *rodZ* and *pgsA*. Therefore, we decided to further characterize the identified sorbic acid-sensitive *rodZ*::mini-Tn*10* strain, as well as a conditional *pgsA* mutant (*pgsA*::*P*_spac_*-pgsA*) strain by testing their susceptibility to other stresses. Since *pgsA* is essential, this strain (MHB001), kindly provided by Kouji Matsumoto (Hashimoto et al., 2013), needs a minimal amount of IPTG to be able to grow. The lowest IPTG concentration which sustained exponential growth similar to that of the WT strain in liquid cultures was 0.1 mM in our exponential conditions. Hereby we wanted to identify whether these genes are involved specifically in lipophilic sorbic acid stress, in weak organic acid stress more in general or in generic stress tolerance. Thus we tested their stress sensitivity for the highly water soluble weak organic acid: acetic acid. Although the side chains of sorbic- and acetic acid differ significantly, both have a similar pK_a_ of 4.76. Additionally we tested osmotic stress by the addition of NaCl, thus investigating a form of stress unrelated to weak organic acid stress.

**FIGURE 2:**
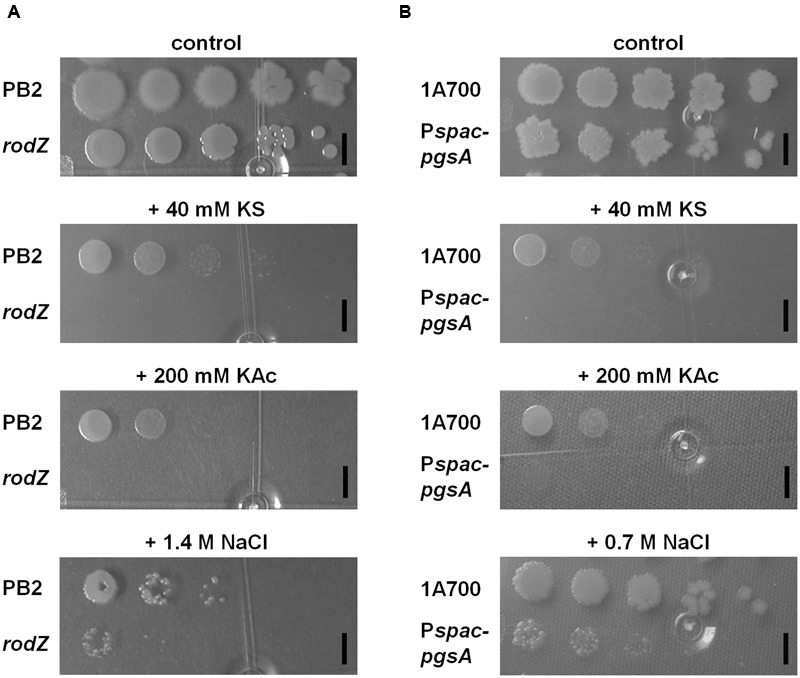
**Characterization of the *rodZ* transposon mutant and a conditional *pgsA* mutant revealed similar (hyper)sensitivity on solid plates for sorbic acid, acetic acid, and NaCl.** WT (PB2), *rodZ*::miniTn*10* (ATB012) **(A)**, and WT (1A700), *pgsA*::P*spac-pgsA* (MHB001) **(B)** were grown exponentially to an OD_600_ of 0.2 when 10-fold dilutions were spotted onto LB plates of pH 6.4 containing no, 40 mM KS, 200 mM KAc, 0.7, or 1.4 M NaCl. Plates were photographed after 24 h of growth at 37°C. The black bars indicate 5 mm.

Next to the previously described sorbic acid-hypersensitivity (**Figure 1**) the results clearly showed also hypersensitivity of the *rodZ* mutant strain for acetic acid stress on plates when compared to the WT strain (**Figure 2A**). No colonies were formed on LB plates of pH 6.4 containing 200 mM potassium acetate (KAc). Additionally, the ability of *rodZ*::mini-Tn*10* cells to form colonies was clearly more inhibited by 1.4 M NaCl than of cells from the parent *B. subtilis* strain. In liquid cultures, the *rodZ* mutant also displayed clear sensitivity toward KAc and NaCl stress (see **Supplementary Figure S2** of the Supporting Information). Interestingly, when grown on solid plates representing the various stress conditions and supplemented with 0.1 mM IPTG, the *pgsA* mutant strain showed a similar stress profile as that of the *rodZ* mutant (**Figure 2B**). Hypersensitivity to sorbic- and acetic acid was observed, and in accordance with previous data, a clear stress sensitivity was detected on plates containing 0.7 M NaCl. The conditional *pgsA* mutant grown in liquid cultures and supplemented with 0.1 mM IPTG also revealed a clear sensitive phenotype toward KS, KAc, and NaCl (see **Supplementary Figure S3** of the Supporting Information). However, the observed phenotypes disappeared when liquid cultures contained 10-fold more (1 mM) IPTG (data not shown).

The strong overlap in the phenotypes of both the *rodZ*::mini-Tn*10* mutant and the conditional *pgsA* mutant and the observation that both genes are co-transcribed suggests that the inactivation of *rodZ* in the transposon mutant gives rise to polar effects. In support of this inference, our RT-PCR data showed ΔΔ*C_T_* values for *pgsA* of up to 5 upon comparing *rodZ*::mini-Tn*10* with control cells using *accA* as reference gene and 3.6 with *rpsM* as reference (See Supplementary Excel Data Sheet S1). Given the observed phenotypes of both mutants and the involvement of PgsA in crucial membrane phospholipid biosynthesis we next assessed the membrane composition in the *rodZ*::mini-Tn*10* and *pgsA*::P*spac-pgsA* strains.

### Phospholipid Composition and Structure in *rodZ::mini-Tn10 and pgsA::Pspac-pgsA*

**FIGURE 3:**
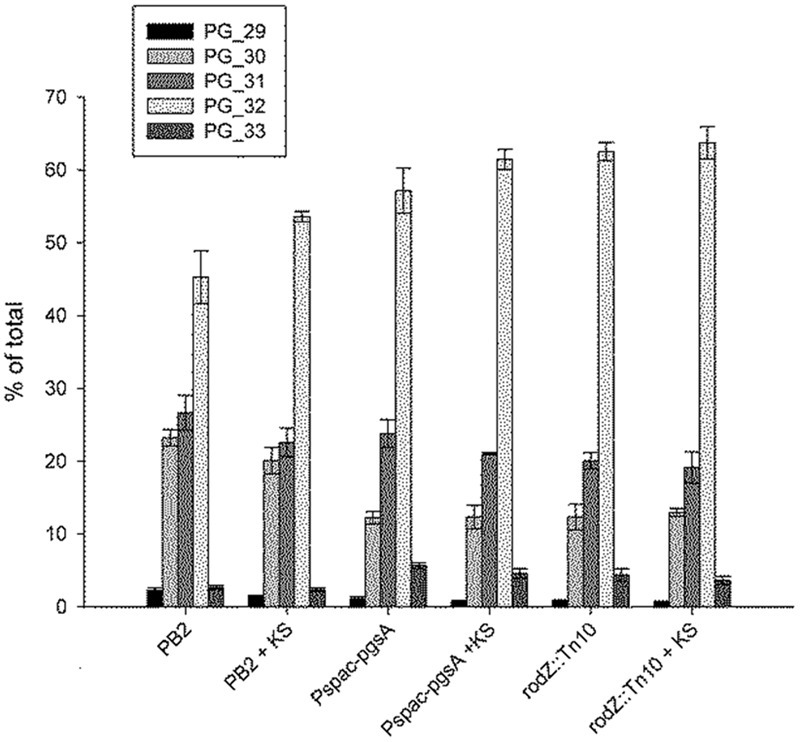
**Increased average acyl-chain length of phosphatidylglycerol (PG) in the *rodZ* transposon and conditional *pgsA* mutant, and increased lengths in the WT strain upon sorbic acid stress.** The acyl chain distribution was measured in exponentially growing cells of the WT (PB2), *pgsA*::P*spac-pgsA* and *rodZ*::mini-Tn*10* mutant strains 45 min after treatment with or without 5 mM KS. The PG acyl chain composition is given as a percentage of the total observed PG levels per strain. The number behind PG in the legend indicates the number of carbon atoms per molecule in the acyl chain.

Our earlier findings suggested that adaptation to sorbic acid would (in part) be by remodeling of the plasma membrane (Ter Beek et al., 2008). We therefore investigated the effect of sorbic acid on the membrane composition of the WT strain, the *rodZ*::mini-Tn*10* mutant, and the *pgsA* conditional mutant (*pgsA*::P*spac*-*pgsA*), as these two mutant strains displayed a similar sensitivity profile. In both the *rodZ* mutant and in the conditional *pgsA* mutant strain (under conditions of minimal *pgsA* expression), the average acyl-chain length of phospholipids was already higher than that of the WT strain when grown without weak organic acid stress. **Figure 3** shows the acyl tail length distribution of PG. Similar results were observed for PG-derived phospholipids (CL and L-PG) and PE (see **Supplementary Figure S4** of the Supporting Information). Upon KS stress, the most prominently observed phospholipid tail length (2⋅16 = 32 carbon atoms) seemed to increase further in the WT (**Figure 3**), corroborating our earlier results (Ter Beek et al., 2008). For the conditional *pgsA* mutant this is less clear, and was not observed in the *rodZ* mutant.

**FIGURE 4:**
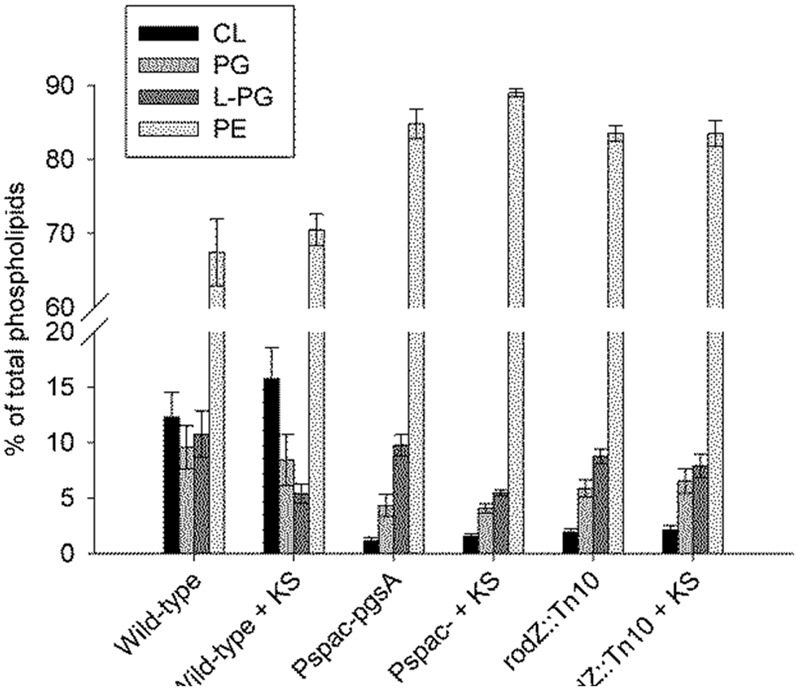
**Lowered cardiolipin (CL) and phosphatidylglycerol (PG) levels in the *rodZ* transposon and conditional *pgsA* mutant, and lowered lysyl-phosphatidylglycerol (L-PG) levels in the WT strain upon sorbic acid stress.** The phospholipid composition was measured in exponentially growing cells of the WT (PB2), *pgsA*::P*spac-pgsA* and *rodZ*::mini-Tn*10* mutant strains 45 min after treatment with or without 5 mM KS.

**FIGURE 5:**
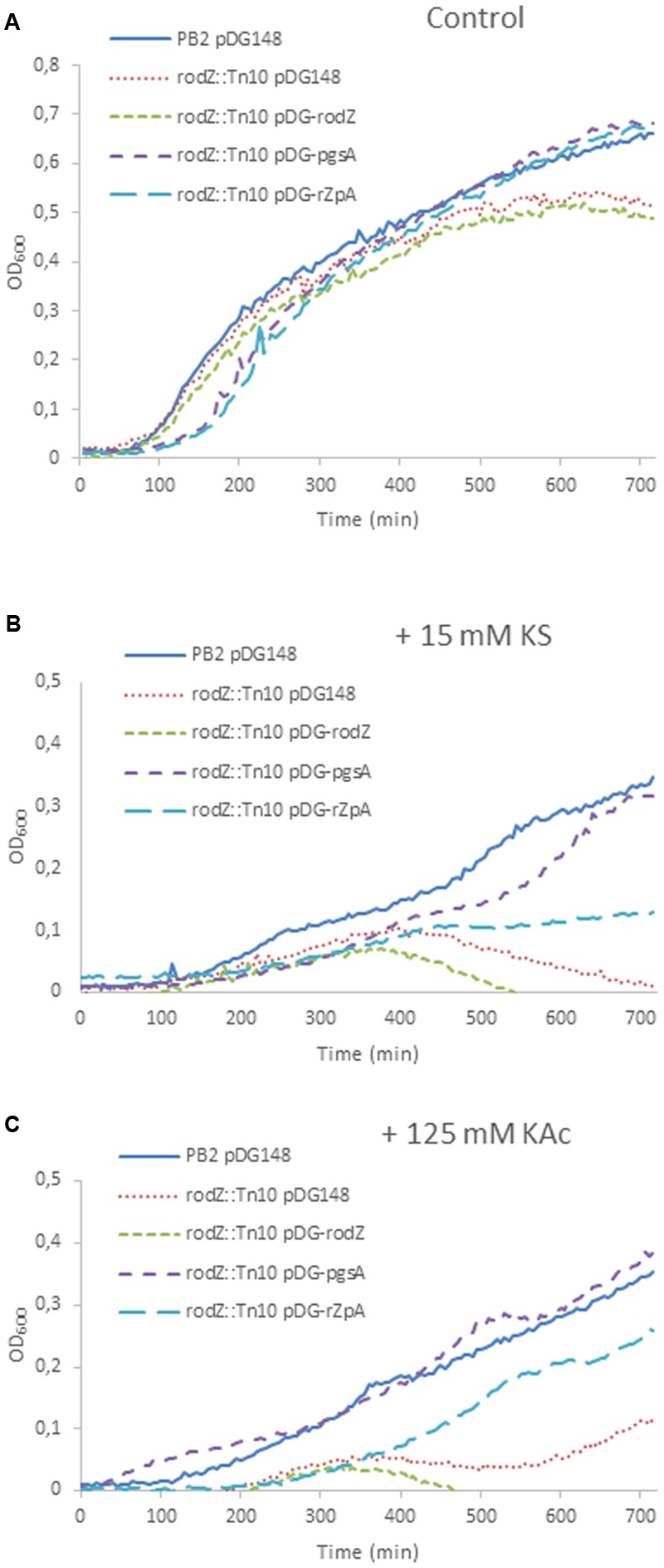
**Rescue of the weak acid phenotypes by overexpression of *pgsA* in the *rodZ* transposon mutant.** Shown are growth curves of *B. subtilis* PB2 pDG148 (solid blue line) and *rodZ*::Tn*10*, transformed with pDG148 (red dotted line), pDG-*rodZ* (green broken line), pDG-*pgsA* (purple broken line), and pDG-*rodZ-pgsA* (light-blue broken line) stressed without **(A)**, and with 15 mM KS **(B)** or 125 mM KAc **(C)**. 1 mM IPTG was added to the pre-cultures of all strains 3 h before the start of the experiments at *t* = 0 min. to induce overexpression.

In terms of the presence of different phospholipid classes the main difference between the WT strain and the *rodZ*::mini-Tn*10* strain under non-stressed conditions is a significant decrease in CL and PG phospholipids in the mutant strain (**Figure 4**). In the conditional *pgsA* mutant similarly lowered levels of CL and PG phospholipids were observed. Consequently, in both mutant strains the relative PE levels were increased when compared to the levels in the WT strain under control conditions. When the WT strain was exposed to sorbic acid, there was a significant drop observed in the relative L-PG levels (**Figure 4**), while the relative content of the other phospholipids was hardly affected. This trend was also observed in the conditional *pgsA* mutant, however, not in the *rodZ* mutant. Compared to the WT strain, the mutants showed a significant shift in the distribution of the acyl chain lengths (longer) (**Figure 3**). Also the mutants seems to have a less negative membrane charge due to the significant reduction of PG (-1) and CL (-2) when compared to the WT (**Figure 5**). Sorbic acid stress does seem to increase the negative net charge on the plasma membrane of WT cells (reduction in L-PG), which may reduce the entry of the sorbate anion. From the data presented it is clear that the phosholipid composition between the two mutants is quite similar, suggesting that the inactivation of *rodZ* may give rise to polar effects and the hypersensitivity of this mutant strain is perhaps caused by lower expression levels of *pgsA*.

### Complementation of the *rodZ*::mini-Tn*10* Mutant

We tried to rescue the stress sensitivity of the *rodZ* transposon mutant by overexpression of *rodZ* and/or *pgsA* by introducing the IPTG-inducible plasmid pDG148 (Stragier et al., 1988) containing *rodZ* and/or *pgsA* under control of a *P*_spac_ promotor. Under control conditions (LB-M, pH = 6.4), the wild-type strain, and mutant strains with pDG148 or pDG-*rodZ* performed equally during early exponential growth. Mutants containing pDG-*pgsA* or pDG-*rodZ-pgsA* displayed a longer lag phase, but ended up with a higher end OD_600_ (**Figure 5A**). Weak acid sensitivity of the *rodZ*::mini-Tn*10* mutant with pDG-*rodZ* (P*spac*-*rodZ*) was not restored (**Figure 5B**). Complementation with *pgsA* via pDG-*pgsA* (P*spac*-*pgsA*) in the *rodZ* mutant reduced KS stress almost completely. Complementation with the pDG-*rodZ-pgsA* plasmid (P*spac*-*rodZ*-*pgsA*) restored some KS sensitivity, but not as much as overexpressing *pgsA* alone (**Figure 5B**). When cultured in the presence of 125 mM KAc stress, the overexpression of RodZ and PgsA from pDG-*rodZ*-*pgsA* or PgsA alone significantly increased the growth rate of the *rodZ*::mini-Tn*10* mutant strain (**Figure 5C**). Induction of pDG-*rodZ* had no effect (**Figure 5C**).

## Discussion

In order to have a direct measure of functional importance for *B. subtilis* weak organic acid stress resistance we decided to opt for the construction and screening of a transposon mutant library for sorbic acid hypersensitive mutants. Sorbic acid (trans-trans-2,4-hexadienoic acid) is a six-carbon unsaturated fatty acid with a pKa of 4.76 and the acid, or its anionic salt, is commonly utilized by the food industry. We chose to screen for sorbic acid-susceptible genes after pre-growing the transposon library first on LB plates for 24 h. We decided to use rich LB medium in our experiments, so that as many as possible mutants were able to grow with relatively normal rate prior to the screen. Longer incubations revealed many more small colonies emerging on the plates. These slow growers on plates were not used in the screen because they evidently already have severe problems growing in control conditions.

Screening of the library for sorbic acid hypersensitivity led to the identification of a uniquely stress sensitive phenotype in which the transposon was inserted into the *rodZ* gene (**Figures 1B,C**). One and the same insertion site was identified in the four discovered clones coming from two independent constructed mutant libraries (**Figure 1A**). The library was made in *B. subtilis* WT strain PB2 using the mini-Tn*10* delivery vector pIC333 and was designed to increase randomness of the sites of insertion (Steinmetz and Richter, 1994). Perhaps that only a specific insertion into the *rodZ* gene of *B. subtilis* resulted in a viable clone with comparable growth rate to the WT strain in control conditions.

Significantly, *rodZ* lies immediately upstream in the genome of the essential *pgsA* gene encoding phosphatidylglycerophosphate synthase (**Figure 1A**). Interestingly, in many other Gram-positive bacteria (e.g., Bacilli, Streptococci, and Stapylococci) *rodZ* and *pgsA* are predicted to be part of the same operon (Ermolaeva et al., 2001; Alm et al., 2005; Alyahya et al., 2009). Our data, showing that a transcript containing both genes can be amplified from *B. subtilis*, corroborates this notion (See **Supplementary Figure S1** of the Supporting Information). Moreover, according to several prediction tools, *B. subtilis* RodZ has one transmembrane domain (amino acids 89 – 113) (Cserzö et al., 1997; Hirokawa et al., 1998) and an Xre-like helix-turn-helix (HTH) motive, commonly seen in DNA binding proteins, in its N-terminal side. Together, these observations initially led us to believe that the RodZ protein might be involved in regulating *pgsA* expression. However, RT-PCR experiments with overexpression of RodZ in the WT strain failed to show any changes in *pgsA* mRNA levels (see **Supplementary Figure S5** of the Supporting Information). HTH motifs also have been shown to function in DNA replication, RNA metabolism, and protein-protein interactions (Aravind et al., 2005). RodZ was shown to co-localize with components of the cytoskeleton and depend on MreB for its localization (Alyahya et al., 2009) and interaction of RodZ with MreB was shown to be specifically with this HTH motif in *Thermotoga maritima* (van den Ent et al., 2010). The mutual functional dependence of RodZ and MreB was reinforced by the observation that loss of RodZ, or at least its N-terminal domain, resulted in aberrant localization of MreB and cessation of its movement (van den Ent et al., 2010; Garner et al., 2011).

Since MreB organization also depends on the membrane potential (Strahl and Hamoen, 2010) and weak organic acids lower the proton gradient (by releasing protons in the cell) and may act in certain cases as uncouplers of the membrane potential (van Beilen et al., 2014), the impact of weak acid stress on cell growth may be partially mediated through membrane perturbation effects on the correct localization of MreB and RodZ containing cell shape determining protein complexes. This in turn could lead to a perturbed localization and functioning of PgsA, exacerbating the sorbic acid sensitivity. PgsA normally localizes primarily to the septal membranes in conjunction with cardiolipin and plays an essential role in cell division (Nishibori et al., 2005).

The observation that *rodZ* and *pgsA* are part of the same transcript as well as our earlier studies (Ter Beek et al., 2008; van Beilen et al., 2014) suggested to us that the membrane plays a crucial role in weak organic acid stress tolerance. Hence we studied the phospholipid composition of the various strains. The *rodZ*::mini-Tn*10* mutant was shown to contain severely lowered levels of PG and CL (**Figure 4**). The average acyl chain length of the remaining phospholipids was increased in the *rodZ* mutant when compared to the WT (**Figure 3**; **Supplementary Figure S4** of the Supporting Information). The same phenomena were observed in the conditional *pgsA*::P*spac- pgsA* mutant that displayed similar sorbic acid-, acetic acid-, and salt-stress sensitivities (**Figure 2**; **Supplementary Figures S2** and **S3** of the Supporting Information). Alterations of the cell membrane lipid composition were also found in response to sorbic acid stress where a pronounced increase in acyl chain length and lowering of L-PG was seen most prominently in WT *B. subtilis* (**Figures 3** and **4**). An increase in chain length stiffens the membrane and lowers its permeability. On the other hand, the induction of the BkdR-regulated genes (involved in the synthesis of precursor molecules for branched-chain fatty acids (Debarbouille et al., 1999) in sorbic acid-stressed cells (Ter Beek et al., 2008) may indicate increased branching in phospholipids and thereby balancing membrane fluidity levels. Interestingly, Lopez and co-workers have shown that salt-stressed cells increase their CL phospholipid levels and decrease both PG and L-PG levels (López et al., 1998), a similar trend observed in KS-stressed cells (**Figure 4**). However, they also measured a clear decrease in branched chain fatty lipids in cultures grown in NaCl. Noteworthy is the observation that both mutants hardly seem to change their membrane composition when stressed with KS (**Figures 3** and **4**). The possible differences in net charge of the membrane between the mutants (significantly reduced PG and CL levels) and the WT may be one explanation for the observed hypersensitivity toward weak organic acid stress. Another explanation might be the inability of the mutants to modify their membrane composition further upon stress, e.g., they cannot adapt further. Besides a potential role for L-PG in weak acid sensitivity, it has strongly been implicated in resistance against cationic antimicrobial peptides (Dunkley et al., 1988; Sohlenkamp et al., 2007; Samant et al., 2009). Alterations in the membrane composition likely pertain to changes in the rate of net proton influx. That is: the rate limiting step for protonophoric uncouplers is the rate with which the anion traverses the liquid-lipid interface (Spycher et al., 2008; Chu et al., 2009; Ter Beek et al., 2014; van Beilen et al., 2014). It is therefore likely that an increase in CL levels in combination with increased acyl-chain length confers increased resistance to uncouplers. On the other hand, this adaptation may conflict with growth at elevated temperatures, but *B. subtilis* may ultimately strive toward homeo-proton permeability (van de Vossenberg et al., 1999). Alternatively, anion build-up has also been implicated as a stress factor caused by weak organic acid preservatives (Russell, 1992). Both of these mechanisms may interfere with proper cytoskeleton function and put stress on the total cell envelop. If one of these players fails to work in concert, the cell is weakend.

Our data presented here on the *rodZ* and the conditional *pgsA* mutant suggest that the phenotypes observed in the *rodZ* transposon mutant might be primarily the result of polar effects on *pgsA* expression. The RT-PCR data on *pgsA* expression levels in the *rodZ*::mini-Tn*10* strain compared to WT PB2 corroborate this conclusion. Finally, overexpression of *pgsA* in the *rodZ*::mini-Tn*10* background stimulated growth of cells cultured in liquid media in the presence of sorbic and acetic acid. However, overexpression of *rodZ* in the *rodZ* transposon mutant did not restore weak acid stress sensitivity to WT levels. These results also indicate that the phenotypes observed in the *rodZ* transposon mutant can almost solely be ascribed to polar effects on *pgsA*.

We present here a direct link between phospholipid synthesis and weak acid sensitivity and propose that PgsA plays an important role in membrane homeostasis and tolerance to weak organic acid stress. Future studies are aimed at assessing the membrane permeation efficacy in *Bacillus* strains with different phospholipid perturbations (Salzberg and Helmann, 2008), by measuring intracellular acidification rates upon addition of various weak organic acids. This can be done with the aid of the pH-sensitive fluorescent protein pHluorin expressed in the lumen of the bacterium. The protocol for this has recently also been established by us in *B. subtilis* (van Beilen and Brul, 2013; van Beilen et al., 2014; Ter Beek et al., 2014).

## Author Contributions

Conceived and designed the experiments: JB, AZ, SB, AB. Performed the experiments: JB, CB, HF, RB, AB. Analyzed the data: JB, CB, AZ, AB. Contributed reagents/materials/analysis tools: WK, FV. Wrote the paper: JB, SB, AB.

## Conflict of Interest Statement

The authors declare that the research was conducted in the absence of any commercial or financial relationships that could be construed as a potential conflict of interest.

## Footnotes

1 http://genolist.pasteur.fr/GenoList/Bacillus

## Supplementary Material

FIGURE S1 ***rodZ* and *pgsA* are co-transcribed.** cDNA synthesized from RNA of PB2 using the reverse primer for *pgsA* (pgsA_Q1_RV) was PCR amplified with the ymfM_Q1_FW and pgsA_Q1_RV primers and run on an agarose gel. M: GeneRuler DNA ladder mix (Fermentas); 1: Genomic DNA of *B. subtilis* WT strain PB2 used as the template (positive control); 2: cDNA made with pgsA_Q1_RV used as the template; 3: No template (primer and RNA control); 4: RNA incubated with RNase A (10 μg/ml) for 20 min at 37°C prior to cDNA reaction (genomic DNA contamination control); 5: No reverse transcriptase control.

FIGURE S2 **Inactivation of *rodZ* leads to increased sensitivity for acetic acid and salt stress in liquid cultures.** Growth curves of WT strain PB2 and the *rodZ*::mini-Tn*10* mutant grown in liquid LB medium of pH 6.4 in control conditions (closed and open circles, respectively) and in the presence of 125 mM KAc **(A)** or 0.7 M NaCl **(B)** (closed downward triangles and open triangles, respectively).

FIGURE S3 **Conditional *pgsA* mutant displays increased sensitivity for sorbic acid, acetic acid, and salt.** Growth curves of WT strain 1A700 and the *pgsA*::P*spac-pgsA* conditional mutant grown in liquid LB medium of pH 6.4 in control conditions (closed and open circles, respectively) and in the presence of 15 mM KS **(A)**, 125 mM KAc **(B)** or 0.7 M NaCl **(C)** (closed downward triangles and open triangles, respectively).

FIGURE S4 **Acyl tail length distributions of phospholipids measured in WT strain PB2, conditional pgsA mutant, and the rodZ transposon mutant, in the absence and presence of 5 mM potassium sorbate (KS).** Acyl chain length distributions of the following phospholipid classes were measured: **(A)** cardiolipin (CL), **(B)** lysyl-phosphatidyl glycerol (L-PG), and **(C)** phosphatidyl glycerol (PE). The number behind the abbreviation indicates the total number of carbon atoms in the acyl chains per molecule.

FIGURE S5 **Overexpression of *rodZ* did not have an effect on *pgsA* expression.** Relative quantification of gene expression using real-time RT-PCR was determined between *B. subtilis* WT strain PB2 carrying pDG148 and pDG-rodZ every 30 min until 2 h after induction. Expression of *rodZ* was induced with 1 mM IPTG at *t* = 0 min in exponentially growing cells. Data were normalized to *accA* expression. The error bars indicate the standard deviation.

## References

[B1] Alm E. J. Huang K. H. Price M. N. Koche R. P. Keller K. Dubchak I. L. (2005). The microbesonline web site for comparative genomics. *Genome Res.* 15 1015–1022. 10.1101/gr.3844805 15998914 PMC1172046

[B2] Alyahya S. A. Alexander R. Costa T. Henriques A. O. Emonet T. Jacobs-Wagner C. (2009). RodZ, a component of the bacterial core morphogenic apparatus. *Proc. Natl. Acad. Sci. U.S.A.* 106 1239–1244. 10.1073/pnas.0810794106 19164570 PMC2633561

[B3] Aravind L. Anantharaman V. Balaji S. Babu M. M. Iyer L. M. (2005). The many faces of the helix-turn-helix domain: transcription regulation and beyond. *FEMS Microbiol. Rev.* 29 231–262. 10.1016/j.femsre.2004.12.008 15808743

[B4] Azukas J. Costilow R. Sadoff H. (1961). Inhibition of alcoholic fermentation by sorbic acid. *J. Bacteriol.* 81 189–194. 13685463 10.1128/jb.81.2.189-194.1961 PMC278985

[B5] Barbe V. Cruveiller S. Kunst F. Lenoble P. Meurice G. Sekowska A. (2009). From a consortium sequence to a unified sequence: the *Bacillus subtilis* 168 reference genome a decade later. *Microbiology* 155 1758–1775. 10.1099/mic.0.027839-0 19383706 PMC2885750

[B6] Bauer B. E. Rossington D. Mollapour M. Mamnun Y. Kuchler K. Piper P. W. (2003). Weak organic acid stress inhibits aromatic amino acid uptake by yeast, causing a strong influence of amino acid auxotrophies on the phenotypes of membrane transporter mutants. *Eur. J. Biochem.* 270 3189–3195. 10.1046/j.1432-1033.2003.03701.x 12869194

[B7] Beales N. (2004). Adaptation of microorganisms to cold temperatures, weak acid preservatives, low pH, and osmotic stress: a review. *Compr. Rev. Food Sci. Food Saf.* 3 1–20. 10.1111/j.1541-4337.2004.tb00057.x 33430556

[B8] Bendezú F. O. Hale C. A. Bernhardt T. G. de Boer P. A. J. (2009). RodZ (YfgA) is required for proper assembly of the MreB actin cytoskeleton and cell shape in *E. coli*. *EMBO J.* 28 193–204. 10.1038/emboj.2008.264 19078962 PMC2637328

[B9] Boylan S. A. Rutherford A. Thomas S. M. Price C. W. (1992). Activation of *Bacillus subtilis* transcription factor sigma B by a regulatory pathway responsive to stationary-phase signals. *J. Bacteriol.* 174 3695–3706. 1592822 10.1128/jb.174.11.3695-3706.1992 PMC206059

[B10] Brul S. Coote P. (1999). Preservative agents in foods. Mode of action and microbial resistance mechanisms. *Int. J. Food Microbiol.* 50 1–17. 10.1016/S0168-1605(99)00072-0 10488839

[B11] Brul S. Ter Beek A. S. (2010). To kill or not to kill Bacilli: opportunities for food biotechnology. *Curr. Opin. Biotechnol.* 21 168–174. 10.1016/j.copbio.2010.03.014 20378332

[B12] Casadaban M. J. Cohen S. N. (1980). Analysis of gene control signals by DNA fusion and cloning in *Escherichia coli*. *J. Mol. Biol.* 138 179–207. 10.1016/0022-2836(80)90283-1 6997493

[B13] Chu S. Hawes J. W. Lorigan G. A. (2009). Solid-state NMR spectroscopic studies on the interaction of sorbic acid with phospholipid membranes at different pH levels. *Magn. Reson. Chem.* 47 651–657. 10.1002/mrc.2444 19444862 PMC4817853

[B14] Cotter P. D. Hill C. (2003). Surviving the acid test: responses of gram-positive bacteria to low pH. *Microbiol. Mol. Biol. Rev.* 67 429–453. 10.1128/MMBR.67.3.429 12966143 PMC193868

[B15] Cserzö M. Wallin E. Simon I. von Heijne G. Elofsson A. (1997). Prediction of transmembrane alpha-helices in prokaryotic membrane proteins: the dense alignment surface method. *Protein Eng.* 10 673–676. 10.1093/protein/10.6.673 9278280

[B16] Davidson P. M. Harrison M. A. (2002). Resistance and adaptation to food antimicrobials, sanitizers, and other process controls. *Food Technol.* 56 69–78.

[B17] Debarbouille M. Gardan R. Arnaud M. Rapoport G. (1999). Role of bkdR, a transcriptional activator of the sigL-dependent isoleucine and valine degradation pathway in *Bacillus subtilis*. *J. Bacteriol.* 181 2059–2066. 10094682 10.1128/jb.181.7.2059-2066.1999 PMC93617

[B18] Dempwolff F. Reimold C. Reth M. Graumann P. L. (2011). *Bacillus subtilis* MreB orthologs self-organize into filamentous structures underneath the cell membrane in a heterologous cell system. *PLoS ONE* 6:e27035 10.1371/journal.pone.0027035 PMC3206058 22069484

[B19] den Blaauwen T. de Pedro M. A. Nguyen-Distèche M. Ayala J. A. (2008). Morphogenesis of rod-shaped sacculi. *FEMS Microbiol. Rev.* 32 321–344. 10.1111/j.1574-6976.2007.00090.x 18291013

[B20] Dunkley E. A. Clejan S. Guffanti A. A. Krulwich T. A. (1988). Large decreases in membrane phosphatidylethanolamine and diphosphatidylglycerol upon mutation to duramycin resistance do not change the protonophore resistance of *Bacillus subtilis*. *Biochim. Biophys. Acta* 943 13–18. 10.1016/0005-2736(88)90341-0 3135835

[B21] Eklund T. (1983). The antimicrobial effect of dissociated and undissociated sorbic acid at different pH levels. *J. Appl. Bacteriol.* 54 383–389. 10.1111/j.1365-2672.1983.tb02632.x 6409875

[B22] Ermolaeva M. D. White O. Salzberg S. L. (2001). Prediction of operons in microbial genomes. *Nucleic Acids Res.* 29 1216–1221. 10.1093/nar/29.5.1216 11222772 PMC29727

[B23] Garner E. C. Bernard R. Wang W. Zhuang X. Rudner D. Z. Mitchison T. (2011). Coupled, circumferential motions of the cell wall synthesis machinery and MreB filaments in *B. subtilis*. *Science* 333 222–225. 10.1126/science.1203285 21636745 PMC3235694

[B24] Gerdes K. (2009). RodZ, a new player in bacterial cell morphogenesis. *EMBO J.* 28 171–172. 10.1038/emboj.2008.287 19194484 PMC2637339

[B25] Hashimoto M. Seki T. Matsuoka S. Hara H. Asai K. Sadaie Y. (2013). Induction of extracytoplasmic function sigma factors in *Bacillus subtilis* cells with defects in lipoteichoic acid synthesis. *Microbiology* 159(Pt. 1) 23–35. 10.1099/mic.0.063420-0 23103977

[B26] Hirokawa T. Boon-Chieng S. Mitaku S. (1998). SOSUI: classification and secondary structure prediction system for membrane proteins. *Bioinformatics* 14 378–379. 10.1093/bioinformatics/14.4.378 9632836

[B27] Houtkooper R. H. Akbari H. van Lenthe H. Kulik W. Wanders R. J. A. Frentzen M. (2006). Identification and characterization of human cardiolipin synthase. *FEBS Lett.* 580 3059–3064. 10.1016/j.febslet.2006.04.054 16678169

[B28] Koppelman C.-M. Aarsman M. E. G. Postmus J. Pas E. Muijsers A. O. Scheffers D.-J. (2004). R174 of *Escherichia coli* FtsZ is involved in membrane interaction and protofilament bundling, and is essential for cell division. *Mol. Microbiol.* 51 645–657. 10.1046/j.1365-2958.2003.03876.x 14731269

[B29] Krulwich T. A. Quirk P. G. Guffanti A. A. (1990). Uncoupler-resistant mutants of bacteria. *Microbiol. Rev.* 54 52–65. 2181259 10.1128/mr.54.1.52-65.1990 PMC372758

[B30] Kunst F. Rapoport G. (1995). Salt stress is an environmental signal affecting degradative enzyme synthesis in *Bacillus subtilis*. *J. Bacteriol.* 177 2403–2407. 7730271 10.1128/jb.177.9.2403-2407.1995 PMC176898

[B31] Livak K. J. Schmittgen T. D. (2001). Analysis of relative gene expression data using real-time quantitative PCR and the 2(-Delta Delta C(T)) method. *Methods* 25 402–408. 10.1006/meth.2001.1262 11846609

[B32] López C. S. Heras H. Ruzal S. M. Sánchez-Rivas C. Rivas E. A. (1998). Variations of the envelope composition of *Bacillus subtilis* during growth in hyperosmotic medium. *Curr. Microbiol.* 36 55–61. 10.1007/s002849900279 9405747

[B33] Maguin E. Duwat P. Hege T. Ehrlich D. Gruss A. (1992). New thermosensitive plasmid for gram-positive bacteria. *J. Bacteriol.* 174 5633–5638. 1324906 10.1128/jb.174.17.5633-5638.1992 PMC206509

[B34] Mascher T. Hachmann A.-B. Helmann J. D. (2007). Regulatory overlap and functional redundancy among *Bacillus subtilis* extracytoplasmic function sigma factors. *J. Bacteriol.* 189 6919–6927. 10.1128/JB.00904-07 17675383 PMC2045236

[B35] Matsumoto K. Kusaka J. Nishibori A. Hara H. (2006). Lipid domains in bacterial membranes. *Mol. Microbiol.* 61 1110–1117. 10.1111/j.1365-2958.2006.05317.x 16925550

[B36] Mols M. van Kranenburg R. Tempelaars M. H. van Schaik W. Moezelaar R. Abee T. (2010). Comparative analysis of transcriptional and physiological responses of *Bacillus cereus* to organic and inorganic acid shocks. *Int. J. Food Microbiol.* 137 13–21. 10.1016/j.ijfoodmicro.2009.09.027 19853945

[B37] Nishibori A. Kusaka J. Hara H. Umeda M. Matsumoto K. (2005). Phosphatidylethanolamine domains and localization of phospholipid synthases in *Bacillus subtilis* membranes. *J. Bacteriol.* 187 2163–2174. 10.1128/JB.187.6.2163-2174.2005 15743965 PMC1064036

[B38] Petit M. A. Bruand C. Jannière L. Ehrlich S. D. (1990). Tn10-derived transposons active in *Bacillus subtilis*. *J. Bacteriol.* 172 6736–6740. 2174858 10.1128/jb.172.12.6736-6740.1990 PMC210787

[B39] Piper P. W. (1999). Yeast superoxide dismutase mutants reveal a pro-oxidant action of weak organic acid food preservatives. *Free Radic. Biol. Med.* 27 1219–1227. 10.1016/S0891-5849(99)00147-1 10641714

[B40] Rasmussen S. Nielsen H. B. Jarmer H. (2009). The transcriptionally active regions in the genome of *Bacillus subtilis*. *Mol. Microbiol.* 73 1043–1057. 10.1111/j.1365-2958.2009.06830.x 19682248 PMC2784878

[B41] Russell J. B. (1992). Another explanation for the toxicity of fermentation acids at low pH: anion accumulation versus uncoupling. *J. Appl. Microbiol.* 73 363–370.

[B42] Salzberg L. I. Helmann J. D. (2008). Phenotypic and transcriptomic characterization of *Bacillus subtilis* mutants with grossly altered membrane composition. *J. Bacteriol.* 190 7797–7807. 10.1128/JB.00720-08 18820022 PMC2583605

[B43] Samant S. Hsu F.-F. Neyfakh A. A. Lee H. (2009). The *Bacillus anthracis* protein MprF is required for synthesis of lysylphosphatidylglycerols and for resistance to cationic antimicrobial peptides. *J. Bacteriol.* 191 1311–1319. 10.1128/JB.01345-08 19074395 PMC2631992

[B44] Sambrook J. Fritsch E. Maniatis T. (1989). *Molecular Cloning: A Laboratory Manual* 2nd Edn. Cold Spring Harbor, NY: Cold Spring Harbor Laboratory Press.

[B45] Sheu C. W. Freese E. (1972). Effects of fatty acids on growth and envelope proteins of *Bacillus subtilis*. *J. Bacteriol.* 111 516–524. 4626502 10.1128/jb.111.2.516-524.1972 PMC251313

[B46] Shiomi D. Sakai M. Niki H. (2008). Determination of bacterial rod shape by a novel cytoskeletal membrane protein. *EMBO J.* 27 3081–3091. 10.1038/emboj.2008.234 19008860 PMC2599877

[B47] Sohlenkamp C. Galindo-Lagunas K. A. Guan Z. Vinuesa P. Robinson S. Thomas-Oates J. (2007). The lipid lysyl-phosphatidylglycerol is present in membranes of *Rhizobium tropici* CIAT899 and confers increased resistance to polymyxin B under acidic growth conditions. *Mol. Plant Microbe Interact.* 20 1421–1430. 10.1094/MPMI-20-11-1421 17977153

[B48] Spycher S. Smejtek P. Netzeva T. I. Escher B. I. (2008). Toward a class-independent quantitative structure–activity relationship model for uncouplers of oxidative phosphorylation. *Chem. Res. Toxicol.* 21 911–927. 10.1021/tx700391f 18358007

[B49] Steinmetz M. Richter R. (1994). Easy cloning of mini-Tn10 insertions from the *Bacillus subtilis* chromosome. *J. Bacteriol.* 176 1761–1763. 8132472 10.1128/jb.176.6.1761-1763.1994 PMC205265

[B50] Stragier P. Bonamy C. Karmazyn-Campelli C. (1988). Processing of a sporulation sigma factor in *Bacillus subtilis*: how morphological structure could control gene expression. *Cell* 52 697–704. 10.1016/0092-8674(88)90407-2 3125985

[B51] Strahl H. Hamoen L. W. (2010). Membrane potential is important for bacterial cell division. *Proc. Natl. Acad. Sci. U.S.A.* 107 12281–12286. 10.1073/pnas.1005485107 20566861 PMC2901462

[B52] Stratford M. Anslow P. A. (1998). Evidence that sorbic acid does not inhibit yeast as a classic “weak acid preservative”. *Lett. Appl. Microbiol.* 27 203–206. 10.1046/j.1472-765X.1998.00424.x 9812395

[B53] Ter Beek A. Keijser B. J. F. Boorsma A. Zakrzewska A. Orij R. Smits G. J. (2008). Transcriptome analysis of sorbic acid-stressed *Bacillus subtilis* reveals a nutrient limitation response and indicates plasma membrane remodeling. *J. Bacteriol.* 190 1751–1761. 10.1128/JB.01516-07 18156260 PMC2258692

[B54] Ter Beek A. Wijman J. G. E. Zakrzewska A. Orij R. Smits G. J. Brul S. (2014). Comparative physiological and transcriptional analysis of weak organic acid stress in *Bacillus subtilis*. *Food Microbiol.* 45(Pt. A) 71–82. 10.1016/j.fm.2014.02.013 25481064

[B55] van Beilen J. W. A. Brul S. (2013). Compartment-specific pH monitoring in *Bacillus subtilis* using fluorescent sensor proteins; a tool to analyse the antibacterial effect of weak organic acids. *Front. Microbiol.* 4:157 10.3389/fmicb.2013.00157 PMC3685010 23785365

[B56] van Beilen J. W. A. Teixeira de Mattos M. J. Hellingwerf K. J. Brul S. (2014). Distinct effects of sorbic acid and acetic acid on the electrophysiology and metabolism of *Bacillus subtilis*. *Appl. Environ. Microbiol.* 80 5918–5926. 10.1128/AEM.01391-14 25038097 PMC4178694

[B57] van de Vossenberg J. L. Driessen A. J. da Costa M. S. Konings W. N. (1999). Homeostasis of the membrane proton permeability in *Bacillus subtilis* grown at different temperatures. *Biochim. Biophys. Acta* 1419 97–104. 10.1016/S0005-2736(99)00063-2 10366675

[B58] van den Ent F. Johnson C. M. Persons L. de Boer P. Löwe J. (2010). Bacterial actin MreB assembles in complex with cell shape protein RodZ. *EMBO J.* 29 1081–1090. 10.1038/emboj.2010.9 20168300 PMC2845281

[B59] White C. L. Kitich A. Gober J. W. (2010). Positioning cell wall synthetic complexes by the bacterial morphogenetic proteins MreB and MreD. *Mol. Microbiol.* 76 616–633. 10.1111/j.1365-2958.2010.07108.x 20233306

[B60] York G. K. Vaughn R. H. (1964). Mechanisms in the inhibition of microorganisms by sorbic acid. *J. Bacteriol.* 88 411–417. 14203358 10.1128/jb.88.2.411-417.1964 PMC277315

