# Supplementary material for: RodZ and PgsA Play Intertwined Roles in Membrane Homeostasis of Bacillus subtilis and Resistance to Weak Organic Acid Stress
Source: Front Microbiol. 2016 Oct 21;7:1633. doi: 10.3389/fmicb.2016.01633 (PMC5073135; doi:10.3389/fmicb.2016.01633)
Supplement: Supplementary file 1 [file Table_1.docx]

**Table S1.** List of primers used in this study.

| **Primer name** | **Sequence (5’-3’)^a^** | **Description** |
| --- | --- | --- |
| pIC333-left | GGCCGATTCATTAATGCAGGG | Left sequencing primer to identify affected DNA by transposon |
| pIC333-right | CGATATTCACGGTTTACCCAC | Right sequencing primer to identify affected DNA by transposon |
| ymfM_F2 | CCCAAGCTT*AAGGAGGAAGCAGGT*ATGTCATTGGATGATCTCCAAGCG | Forward primer for *rodZ* including ribosome binding site |
| ymfM_RV3 | ACGCGTCGACCCCTCCAATTTCATACTCGGACG | Reverse primer for *rodZ* |
| pgsA_FW | CCCAAGCTT*AAGGAGGAAGCAGGT*ATGTTTAACTTACCAAATAAAATCACACTAGC | Forward primer for *pgsA* including ribosome binding site |
| pgsA_RV | ACGCGTCGACCTTTCTTAGTTAGATGTTTTTAACGCTTCCCAATTTTTAGAG | Reverse primer for *pgsA* |
| ymfM-Q1F | GCCAAAGCCAGCATCTAAGG | Forward primer to determine *rodZ* expression |
| ymfM-Q1R | CACGCCGAGAATAACAAGTATTGT | Reverse primer to determine *rodZ* expression |
| pgsA-Q1F | TTGCGGACAAACTGCTCGTA | Forward primer to determine *pgsA* expression |
| pgsA-Q1R | TGGAGCGAGATCAAATTGAACA | Reverse primer to determine *pgsA* expression |
| accA-F | GGGACGATGAAGCCATTGTC | Forward primer to determine *accA* expression |
| accA-R | CTGATGCCCGATTACCGTTAC | Reverse primer to determine *accA* expression |

^a^Restriction sites are underlined and the Shine-Dalgarno region is shown in italics.

**Supplementary excel Data Sheet S1**

Supplementary excel data sheet of *rodZ*(*ymfM*) and *pgsA* Q-PCR data including the ΔΔ*C_T_* data according to Litvak and Schmittgen (2001) for a comparison of the relative gene expression between *ymfM* (*rodZ*::miniTn*10*) cells and the WT PB2 strain.
