# Supplementary figures and images for: RodZ and PgsA Play Intertwined Roles in Membrane Homeostasis of Bacillus subtilis and Resistance to Weak Organic Acid Stress
Source: Front Microbiol. 2016 Oct 21;7:1633. doi: 10.3389/fmicb.2016.01633 (PMC5073135; doi:10.3389/fmicb.2016.01633)

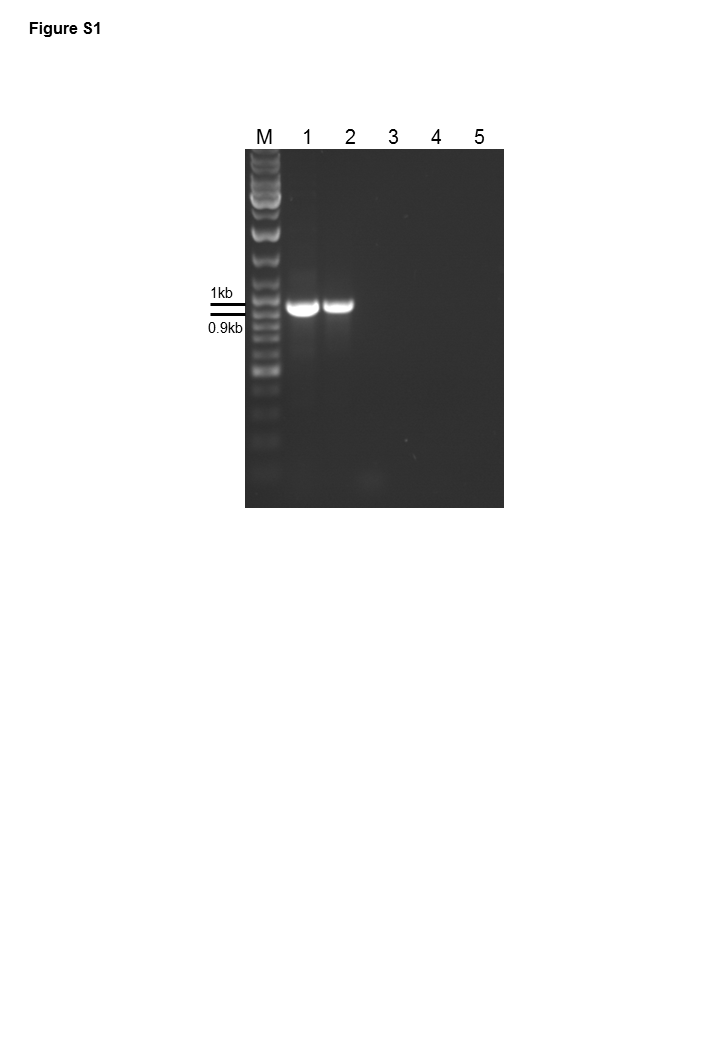

Supplement: FIGURE S1 — rodZ and pgsA are co-transcribed. cDNA synthesized from RNA of PB2 using the reverse primer for pgsA (pgsA_Q1_RV) was PCR amplified with the ymfM_Q1_FW and pgsA_Q1_RV primers and run on an agarose gel. M: GeneRuler DNA ladder mix (Fermentas); 1: Genomic DNA of B. subtilis WT strain PB2 used as the template (positive control); 2: cDNA made with pgsA_Q1_RV used as the template; 3: No template (primer and RNA control); 4: RNA incubated with RNase A (10 μg/ml) for 20 min at 37°C prior to cDNA reaction (genomic DNA contamination control); 5: No reverse transcriptase control. [file Image_1.tif]

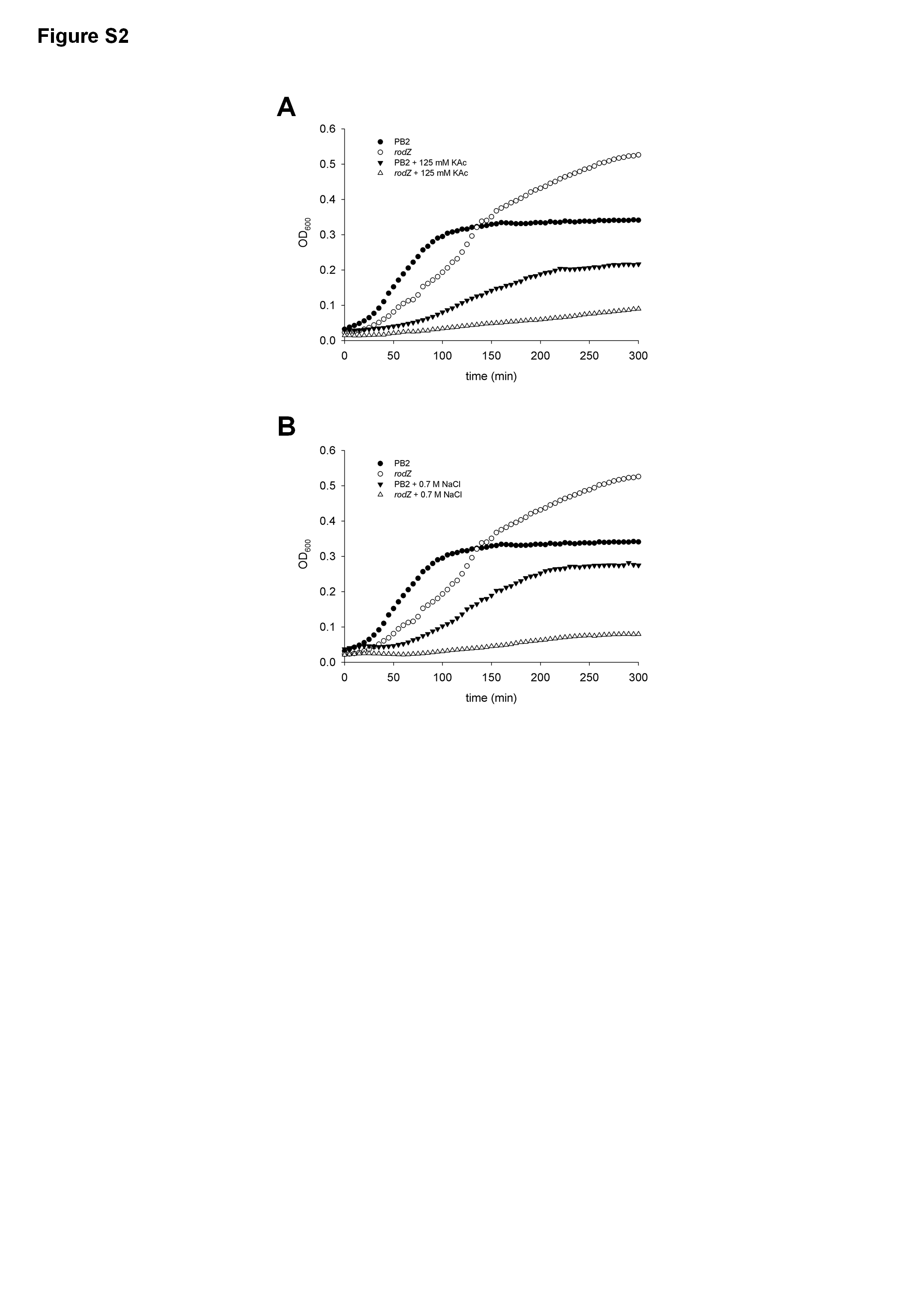

Supplement: FIGURE S2 — Inactivation of rodZ leads to increased sensitivity for acetic acid and salt stress in liquid cultures. Growth curves of WT strain PB2 and the rodZ::mini-Tn10 mutant grown in liquid LB medium of pH 6.4 in control conditions (closed and open circles, respectively) and in the presence of 125 mM KAc (A) or 0.7 M NaCl (B) (closed downward triangles and open triangles, respectively). [file Image_2.tif]

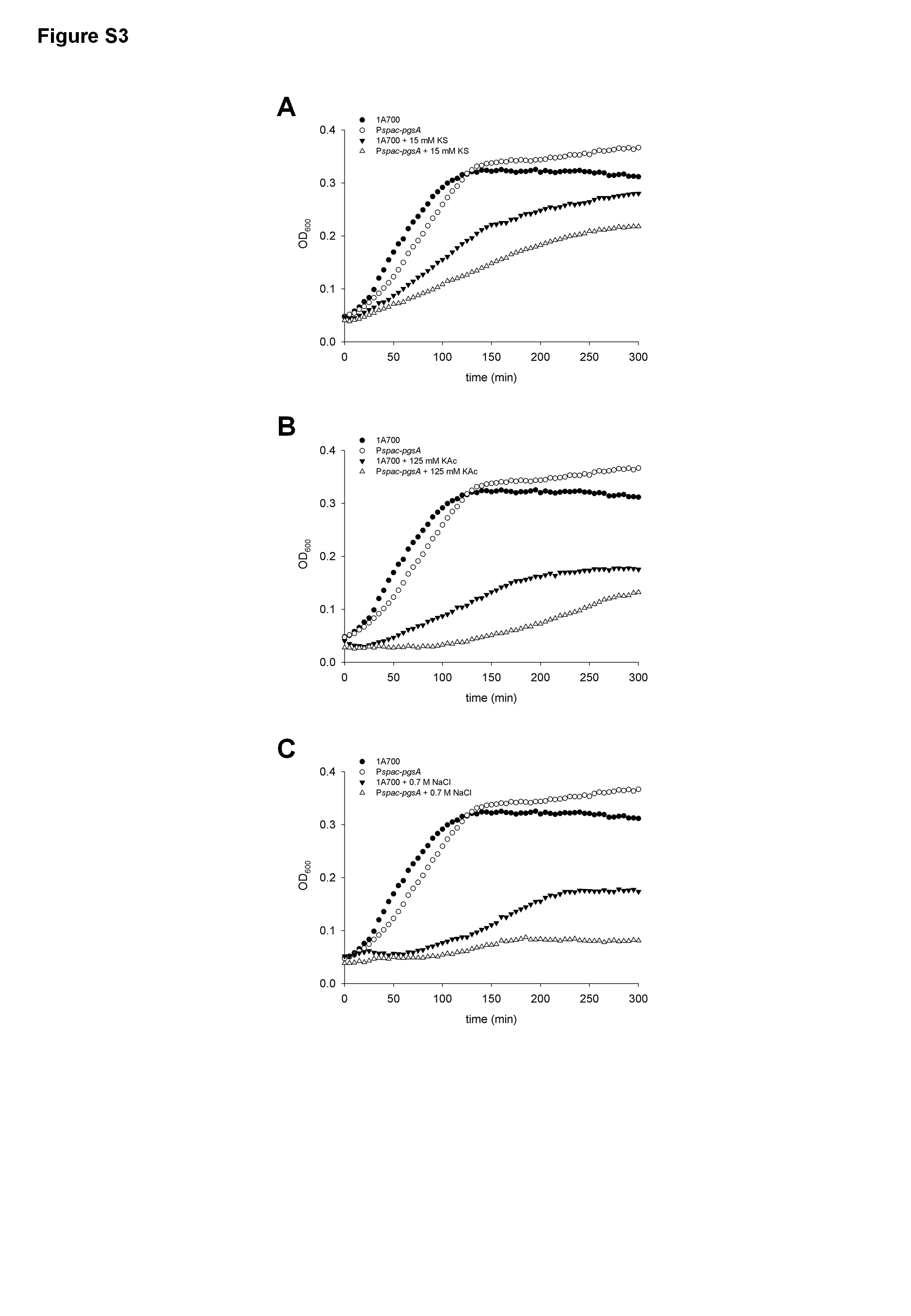

Supplement: FIGURE S3 — Conditional pgsA mutant displays increased sensitivity for sorbic acid, acetic acid, and salt. Growth curves of WT strain 1A700 and the pgsA::Pspac-pgsA conditional mutant grown in liquid LB medium of pH 6.4 in control conditions (closed and open circles, respectively) and in the presence of 15 mM KS (A), 125 mM KAc (B) or 0.7 M NaCl (C) (closed downward triangles and open triangles, respectively). [file Image_3.tif]

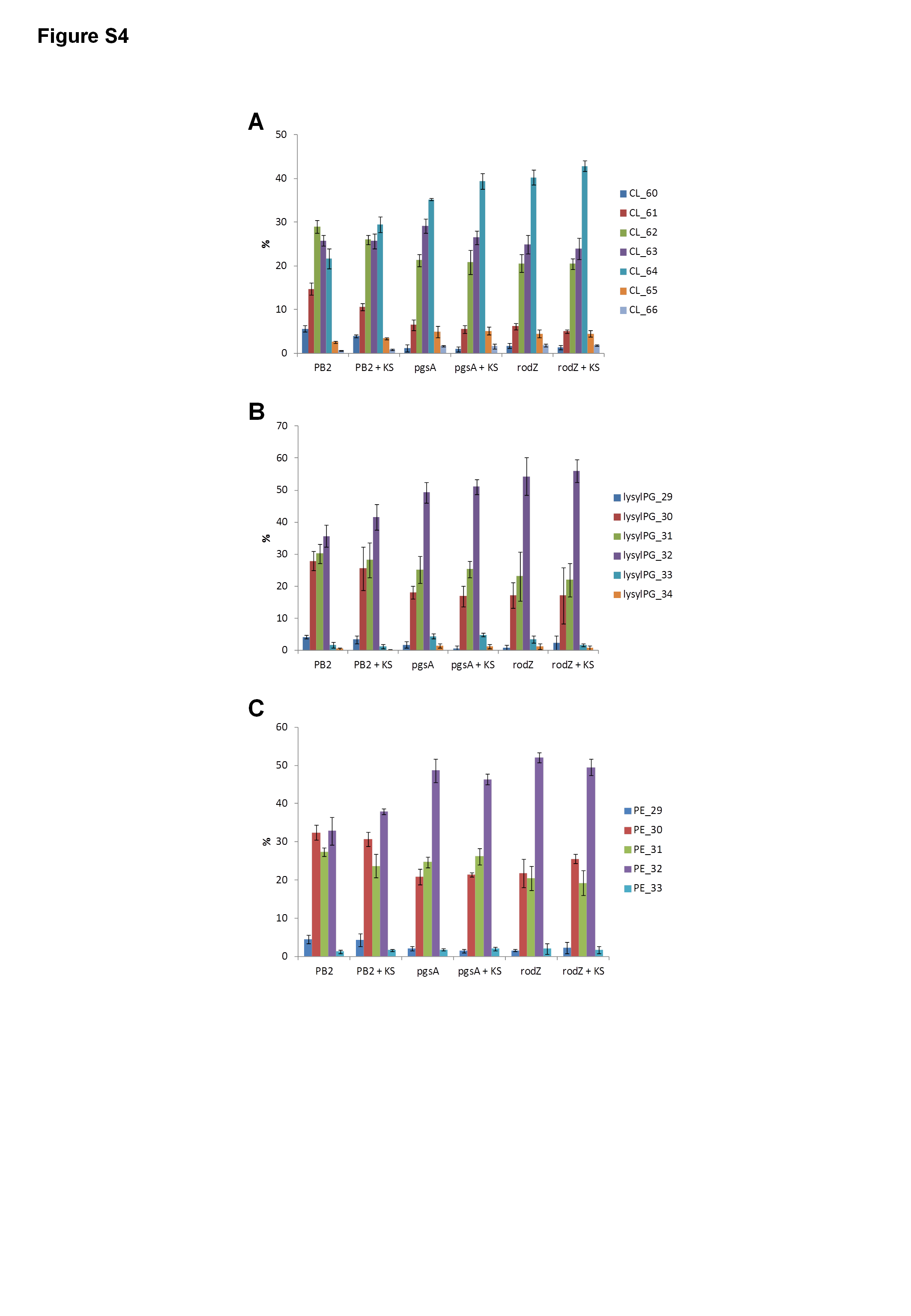

Supplement: FIGURE S4 — Acyl tail length distributions of phospholipids measured in WT strain PB2, conditional pgsA mutant, and the rodZ transposon mutant, in the absence and presence of 5 mM potassium sorbate (KS). Acyl chain length distributions of the following phospholipid classes were measured: (A) cardiolipin (CL), (B) lysyl-phosphatidyl glycerol (L-PG), and (C) phosphatidyl glycerol (PE). The number behind the abbreviation indicates the total number of carbon atoms in the acyl chains per molecule. [file Image_4.tif]

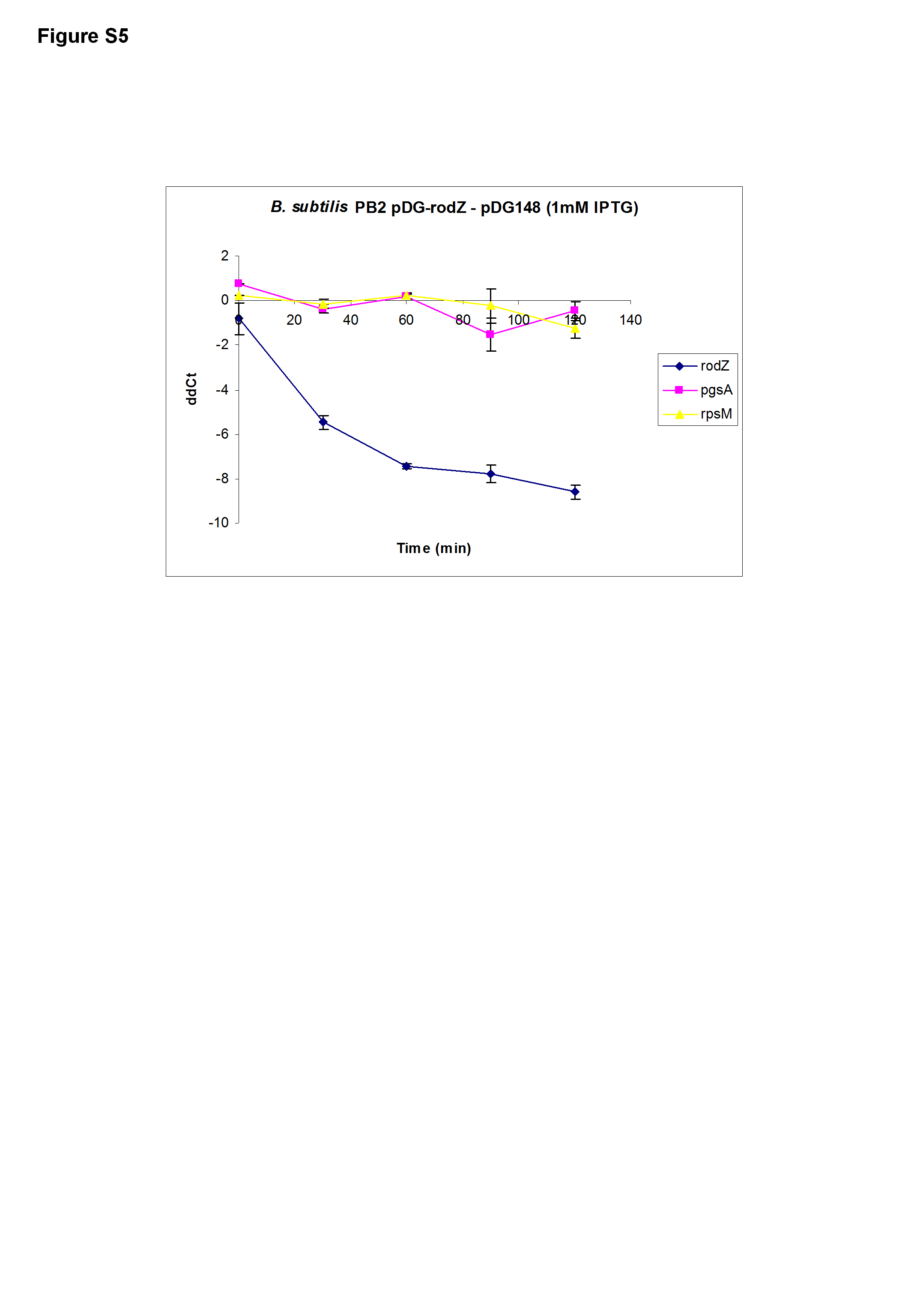

Supplement: FIGURE S5 — Overexpression of rodZ did not have an effect on pgsA expression. Relative quantification of gene expression using real-time RT-PCR was determined between B. subtilis WT strain PB2 carrying pDG148 and pDG-rodZ every 30 min until 2 h after induction. Expression of rodZ was induced with 1 mM IPTG at t = 0 min in exponentially growing cells. Data were normalized to accA expression. The error bars indicate the standard deviation. [file Image_5.tif]
